# Effect of Heat Accumulation on Femtosecond Laser Reductive Sintering of Mixed CuO/NiO Nanoparticles

**DOI:** 10.3390/mi9060264

**Published:** 2018-05-28

**Authors:** Mizue Mizoshiri, Kenta Nishitani, Seiichi Hata

**Affiliations:** 1 Department of Mechanical Engineering, Nagaoka University of Technology, 1603-1, Kamitomioka, Nagaoka, Niigata 940-2188, Japan; 2 Department of Micro-Nano Mechanical Science and Engineering, Nagoya University, Furo-cho, Chikusa, Nagoya, Aichi 464-8603, Japan; nishitani.kenta@a.mbox.nagoya-u.ac.jp (K.N.); hata@mech.nagoya-u.ac.jp (S.H.)

**Keywords:** direct writing, femtosecond laser, reductive sintering, thermoelectric film, Cu-Ni alloy, micropatterns, printing

## Abstract

Direct laser-writing techniques have attracted attention for their use in two- and three-dimensional printing technologies. In this article, we report on a micropatterning process that uses femtosecond laser reductive sintering of mixed CuO/NiO nanoparticles. The writing speed, laser fluence, and incident total energy were varied to investigate the influence of heat accumulation on the micropatterns formed by these materials. Heat accumulation and the thermal history of the laser irradiation process significantly affected the material composition and the thermoelectric properties of the fabricated micropatterns. Short laser irradiation durations and high laser fluences decrease the amount of metal oxide in the micropatterns. Selective fabrication of p-type and n-type thermoelectric micropatterns was demonstrated to be possible with control of the reduction and reoxidization reactions through the control of writing speed and total irradiation energy.

## 1. Introduction

Direct laser-writing techniques have attracted attention in printing technologies. For example, three-dimensional (3D) printing, which is known as additive manufacturing, has been used in the fabrication of 3D bulk metal structures. In the process, raw metal powders are sintered and melted with heat from a laser in an inert atmosphere such as a vacuum or an inert gas. To date, structures made of many metals and alloys, such as Cu, Fe-Ni, and Ti-6Al-4V, have been achieved by selectively sintering and melting powders of the materials [1,2,3,4]. However, it is difficult to scale these processes for producing metal microstructures because fine metal powders oxidize too easily.

To fabricate 2D and 3D metal microstructures, metals and alloys can be printed from metal nanoparticle (NP) inks and metal oxide NP solutions. For example, metal NP inks with Au, Ag, or Cu NPs mixed with dispersants have been selectively sintered using lasers in ambient atmosphere [5,6,7]. Neodymium-doped yttrium aluminum garnet (Nd/YAG) lasers operating with the wavelength of 532 nm are particularly effective for Cu sintering because Cu exhibits higher absorbance for this wavelength than it does for infrared light. Flexible displays composed of such metal electrodes have been fabricated using the printing techniques.

Metal oxide NP solutions with NPs of CuO, Cu_2_O, and NiO, a reducing agent, and a dispersant, have been used for laser reductive sintering [8,9,10,11]. A CuO NP solution composed of CuO NPs, ethylene glycol (EG) as the reductant, and polyvinylpyrrolidone (PVP) as the dispersant, was reduced and sintered using continuous-wave and nanosecond lasers at a wavelength of 1070 nm [8]. The advantage of the process is that the CuO NP solution considerably absorbs the laser light since the band gap of CuO is 1.2 eV (wavelength 1033 nm). Two-dimensional Cu micropatterns have been formed on glass substrates and polyimide films using reductive sintering [8]. In another work, Cu_2_O NP solutions were prepared by mixing of Cu_2_O NPs, 2-propanol, and PVP. When a laser beam is focused onto the Cu_2_O NP solutions, formic acid is generated by the thermal reaction of 2-propanol and PVP. Then, the formic acid reduces the Cu_2_O to Cu [9]. Ni micropatterns have also been fabricated by laser reductive sintering of NiO NPs [10,11]. In this process, solutions of NiO NPs and toluene are reduced by irradiation with a 514.5-nm continuous wave laser to fabricate 2D and Ni patterns.

We have also developed a process for femtosecond laser reductive sintering of metal oxide NPs [12,13,14]. Femtosecond laser pulses are effective for controlling the reduction and reoxidization of the micropatterns. For example, Cu-rich and Cu_2_O-rich micropatterns can be formed selectively by controlling laser irradiation conditions such as the writing speed [12]. We have used this process to fabricate Cu/Cu_2_O composite micro-thermistors. Furthermore, NP solutions with the mixtures of NiO/Cr and CuO/NiO have enabled us to form Cu-Ni alloys and Ni/Cr-O composite micropatterns, respectively [13,14]. Ni/Cr-O microgears were successfully fabricated, and they could be moved by controlling an external magnetic field. Cu-Ni and Cu_2_O/NiO micropatterns exhibit n-type and p-type thermoelectric properties, respectively [14]. In addition, we applied the selective micropatterning process to demonstrate the performance of thermocouples. The selective fabrication of Cu-Ni and Cu_2_O/NiO micropatterns is possible by controlling the laser-writing speed to be as high as 1–20 mm/s, which can lead to problems in the fabrication process. The effects of heat accumulation on the reduction and reoxidization reactions are complex because the balance of the reduction and reoxidization reactions is determined by the chemical potentials and activation energies of the metals (Cu, Ni) and O. Micropatterns are typically formed by raster scanning of a focused laser beam. Therefore, such high writing speeds are not ideal since the scale of the micropatterns is limited by the significant effects to the material around the edges of the laser-irradiated area.

This article reports our investigation of the effects of heat accumulation on micropatterns formed through femtosecond laser reductive sintering of CuO/NiO mixed NPs. The details of the patterning properties were evaluated at writing speeds including below 1 mm/s. The crystal structures and metal oxide composites of the fabricated micropatterns were subsequently examined. Then, the Seebeck coefficients of the micropatterns were calculated. Finally, we fabricated thermocouples to demonstrate the process’s effectiveness for precisely fabricating fine micropatterns with controllable thermoelectric properties.

## 2. Materials and Methods

CuO/NiO NP solution was prepared by mixing CuO NPs (Sigma Aldrich, St. Louis, MO, USA, diameter < 50 nm), NiO NPs (Sigma Aldrich, St. Louis, MO, USA, diameter < 50 nm), PVP (Sigma Aldrich, St. Louis, MO, USA, M_w_~10,000), and EG (Sigma Aldrich, St. Louis, MO, USA) using ultrasonic agitation. The concentrations of CuO NPs, NiO NPs, PVP, and EG were 36.9 wt %, 23.1 wt %, 13 wt %, and 27 wt %, respectively. Then, the CuO/NiO NP solution was spin-coated on 1-mm-thick glass substrates. The thickness of the coated film was ~10 µm. Direct writing was subsequently performed in air using a femtosecond laser-writing system (Photonic Professional GT, Nanoscribe GmbH, Eggenstein-Leopoldshafen, Germany). Femtosecond laser pulses operating at a wavelength of 780 nm, repetition rate of 80 MHz, and pulse duration of 120 fs were focused onto the surface of the films using an objective lens with numerical aperture of 0.75. The focused beam diameter was ~1.3 µm. The laser polarization was linear. Micropatterns were formed by scanning the focused laser pulses using an x-y mechanical stage. The writing speed was varied in the range of 100–5000 µm/s. The micropatterns were written by raster scanning of the laser focal spot at a pitch of 10 µm, which was determined by considering the minimum line width of ~10 µm. Finally, residual non-irradiated NPs were removed by rinsing the substrates in EG and ethanol.

The morphology of the micropatterns was observed using a scanning electron microscope (SEM, Hitachi High Technologies, Tokyo, Japan, TM3030Plus). These observations were performed in the SEM’s low vacuum mode without coating of the electrical thin films to reduce their electrical charge. The crystal structures of the fabricated micropatterns were examined using an imaging plate X-ray diffraction (XRD) apparatus (Rigaku, Tokyo, Japan, Rint Rapid-S diffractometer) with a collimated beam diameter of 0.3 mm. The incident angle was fixed to 20°. The oxide materials included in the micropatterns, such as CuO, Cu_2_O, and NiO, were evaluated via Raman spectrometry using a 532-nm laser.

The Seebeck coefficient *S* of each micropattern was estimated by measuring both the temperature difference Δ*T* between the fabricated micropatterns and the voltage *V* generated by that temperature difference. Infrared thermography (Nippon Avionics, Tokyo, Japan, Thermo Shot F30) was used to assess the temperature difference. The voltage generated was measured with a multimeter (Keysight Technology, Santa Rosa, CA, USA, Truevolt series 34465A). The Seebeck coefficient *S* was defined as *V*/Δ*T*.

## 3. Results

In preliminary tests, the crystal structures of the fabricated micropatterns were clarified with XRD analysis. The oxide compositions of the micropatterns were also measured using Raman spectroscopy. Then, we measured the micropattern responses with various writing conditions such as the writing speed, incident total energy, and laser fluence to evaluate effects of heat accumulation on the fabricated micropatterns.

### 3.1. Micropatterns at Various Writing Speed

**Figure 1:**
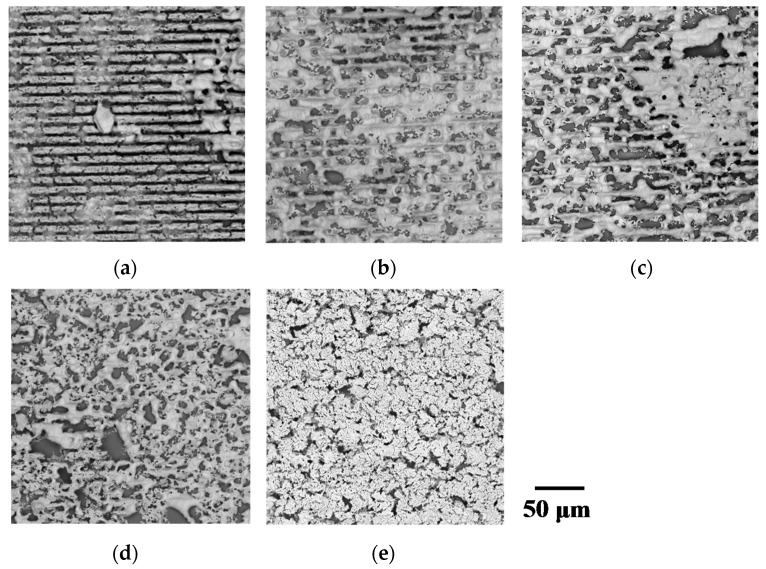
SEM images of micropatterns fabricated at writing speeds of (**a**) 100 µm/s, (**b**) 500 µm/s, (**c**) 1000 µm/s, (**d**) 3000 µm/s, and (**e**) 5000 µm/s, respectively.

First, the morphologies of the fabricated micropatterns were observed using SEM images. Figure 1a–e show the surface morphologies of the micropatterns fabricated at a laser fluence of 0.059 J/cm^2^ and writing speeds of 100, 500, 1000, 3000, and 5000 µm/s, respectively. Lines from the laser scanning appeared in the form of grooves at the low writing speeds of 100–1000 µm/s. The surfaces were partially melted at these low writing. We consider that irradiating laser pulses with a high repetition rate induced the heat accumulation that ablated the material at low writing speeds However, micropatterns with somewhat uniform surfaces were formed at the writing speed of 5000 µm/s. In this case, the raw NPs seem to have been sintered instead of melted.

**Figure 2:**
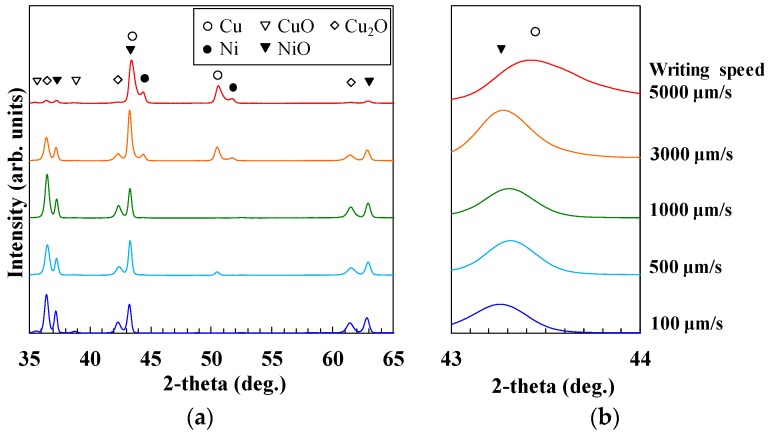
(**a**) X-ray diffraction (XRD) spectra of micropatterns, (**b**) enlargement of these spectra from 43–44 degrees.

Figure 2 shows XRD spectra recorded from the micropatterns. Metal or metal oxide composite micropatterns were obtained, depending on the writing speed. With the fast writing speed of 5000 µm/s, XRD intensity peaks corresponding to the oxides were weak. On the other hand, intense XRD peaks corresponding to Cu_2_O and NiO appeared with low writing speeds of 100–3000 µm/s. CuO was clearly generated only at the writing speed of 100 µm/s. The broad spectra were observed between the peaks of Cu and Ni in Figure 2a. In addition, the peak shift was also observed at 43°–44° in the micropatterns written at 5000 µm/s in Figure 2b. These results suggest the possibility of the generation of Cu-Ni alloy or metal oxides.

**Figure 3:**
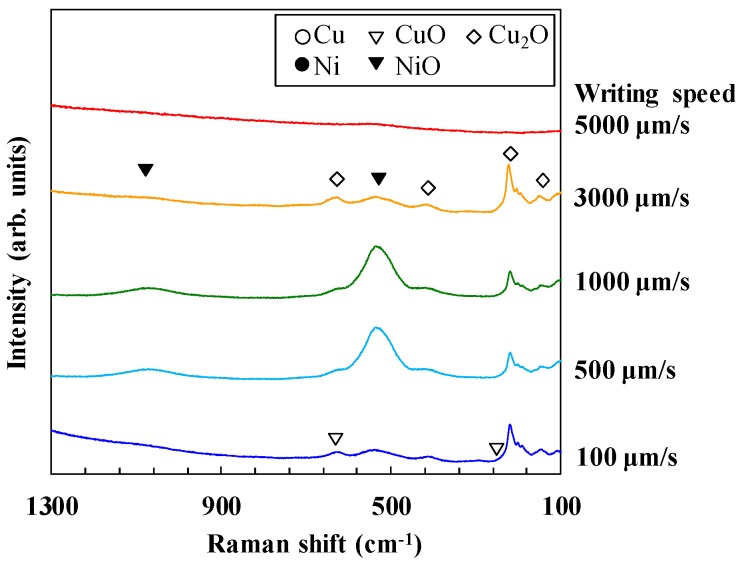
Raman shifts of the fabricated micropatterns.

The separations of the diffraction peaks for NiO and Cu at ~43.3° are difficult to distinguish. To examine the composition ratio of the metal and metal oxide in the micropatterns, Raman spectra of the fabricated micropatterns were evaluated, and the evaluation results are shown in Figure 3. No peak appeared with the writing speed of 5000 µm/s, suggesting that metal oxides were not included in the micropatterns. However, obvious peaks corresponding to NiO appeared at the writing speeds of 500 and 1000 µm/s. By considering the XRD and Raman spectra of the micropatterns together, we found that the XRD peak shift at 43°–44° in the micropatterns written at 5000 µm/s indicates the generation of Cu-Ni alloy because the Raman spectra show that the micropatterns did not include metal oxides.

The Seebeck coefficients of the micropatterns are listed in Table 1. p-type micropatterns were obtained with the low writing speeds of 100–500 µm/s. This result is consistent with XRD and Raman spectra results that indicate the generation of Cu_2_O and NiO, which is a p-type material. n-type micropatterns were obtained at the fast writing speed of 5000 µm/s. XRD and Raman spectra results indicate the generation of low amounts of metal oxides such as Cu_2_O, CuO, and NiO, and high amounts of Cu-Ni alloy in the n-type thermoelectric material. The n-type thermoelectric properties generated with the writing speed of 5000 µm/s are owing to the presence of Cu-Ni alloys.

### 3.2. Micropatterns Formed with Various Laser Fluences

Next, the effects of the laser fluence on the micropatterns were investigated while the total incident energy kept constant. The writing speed was decided depending on the laser fluence to maintain constant total incident energy across these trials. Table 2 lists the laser irradiation conditions.

**Figure 4:**
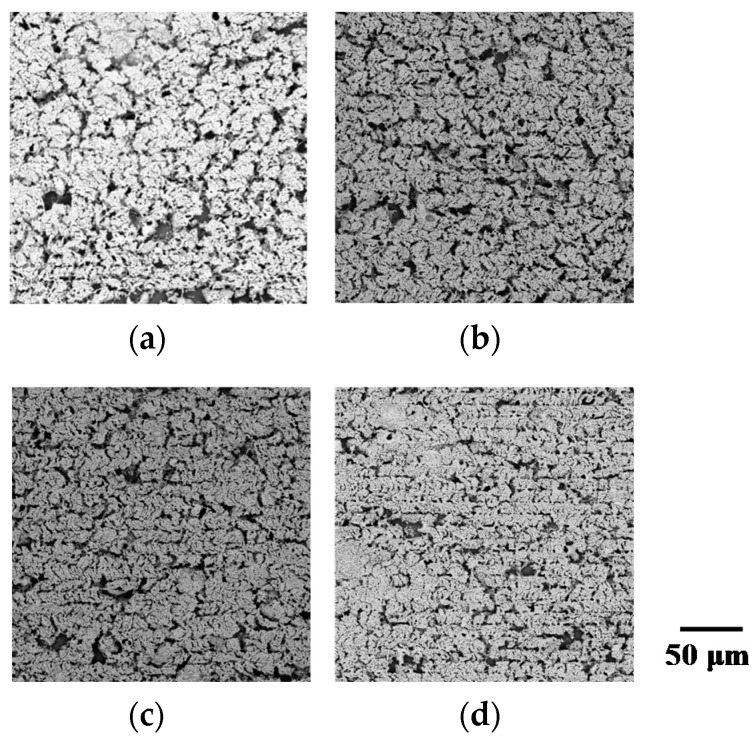
SEM images of the fabricated micropatterns at laser fluences of (**a**) 0.059 J/cm^2^, (**b**) 0.047 J/cm^2^ µm/s, (**c**) 0.035 J/cm^2^, and (**d**) 0.024 J/cm^2^.

Figure 4a–d show SEM images of the surface morphology of the micropatterns fabricated with laser fluence 0.024–0.059 J/cm^2^. The laser fluence of 0.012 J/cm^2^ did not form any micropatterns because they were washed from the substrate during the rinsing step that removed non-irradiated NPs. Compared to the different incident total energies in Figure 1, all the micropatterns have similar surfaces. They seem to have been formed by the sintering of the NPs without any obvious melting. This result indicates that the total incident energy determined the surface morphology of the micropatterns.

**Figure 5:**
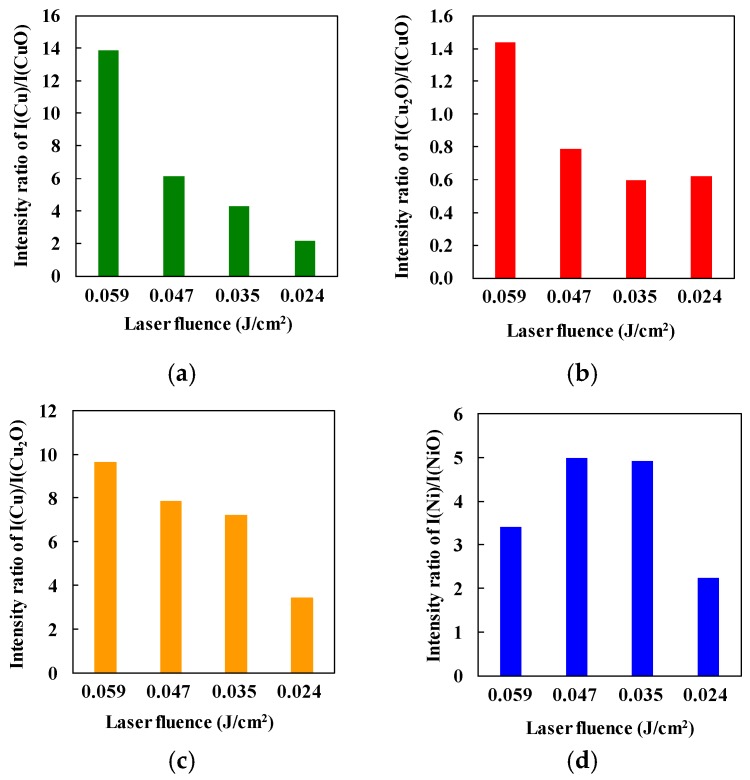
Intensity ratios of (**a**) I(Cu)/I(CuO), (**b**) I(Cu_2_O)/I(CuO), (**c**) I(Cu)/I(Cu_2_O), and (**d**) I(Ni)/I(NiO).

To evaluate the proportions of metals and metal oxides included in the micropatterns, the intensity ratios of the XRD spectra I(Cu_(111)_/CuO_(111)_) for generating Cu, I(Cu_2_O_(111)_/CuO_(111)_) for generating Cu_2_O, I(Cu_(111)_/Cu_2_O_(111)_) for evaluating the degree of CuO reduction, and I(Ni_(111)_/NiO_(111)_) for generating Ni, were calculated. Figure 5a–d show the intensity ratios for each micropattern. The generation of both Cu and Cu_2_O increased with increasing laser fluence, as shown in Figure 4a,b. In addition, Cu generation increased with increasing laser fluence. These results indicate that high laser fluence is important to reduce the CuO/NiO NPs entirely. High and low laser fluence levels are effective for generation of Cu and Cu_2_O, respectively.

However, Ni generation in the micropatterns at the laser fluence of 0.059 J/cm^2^ was less than that achieved with the fluence of 0.047 J/cm^2^. This difference suggests that the Ni was reoxidized at the laser fluence of 0.059 J/cm^2^ because Ni is oxidized more easily than Cu. Therefore, the high generation of Cu may be induced by the contributing of Ni acting as a reductant.

The Seebeck coefficients of the micropatterns are listed in Table 3. All the micropatterns exhibited n-type thermoelectric properties. The largest negative value was obtained at the laser fluence of 0.059 J/cm^2^, that is consistent with the small amount of generation of the p-type oxides such as Cu_2_O and NiO.

### 3.3. Effect of Heat Accumulation on Micropatterning

**Figure 6:**
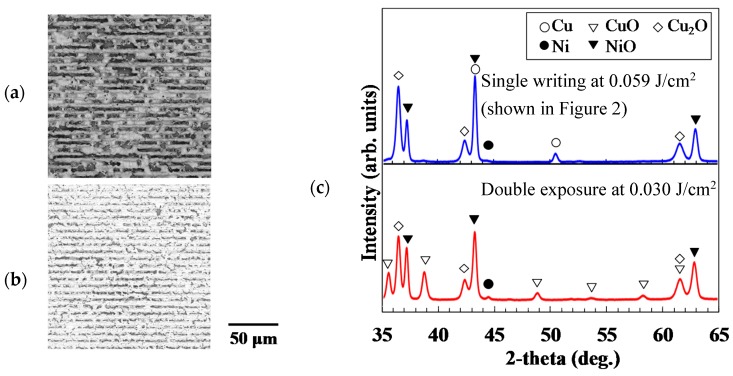
SEM images of the surfaces of micropatterns fabricated with (**a**) single exposure at 0.059 J/cm^2^ and (**b**) double exposure at 0.030 J/cm^2^; (**c**) XRD spectra of the micropatterns.

To evaluate the effect of heat accumulation on the micropatterning, single- and double-exposed micropatterns were fabricated. The laser fluences were chosen as 0.059 J/cm^2^ for single exposure and 0.030 J/cm^2^ for double exposure. The writing speed was fixed at 500 µm/s. In the double exposure, firstly the micropattern was written by raster scanning. Then, the second scanning was performed on the first exposed area. The duration between the end of the first exposure and the start of the second exposure was several few seconds, which was expected to be enough to cool the heated materials at the first exposure. Figure 6a,b show SEM images of the surface morphology of these micropatterns. Deep grooves appeared with single writing at high laser fluence. These results indicate that the high laser fluence caused rapid heating and ablation where the laser was irradiated the material. XRD spectra of the micropatterns are shown in Figure 6c. The generation of metal oxide was higher with double exposure at 0.030 J/cm^2^ than that with single writing at 0.059 J/cm^2^. Compared to the case of double exposure with a low laser fluence, the maximum temperature is high in the case of single writing with a high laser fluence. Therefore, the reduction and sintering of the metal oxide NPs seems to be caused in short duration, preventing the reoxidation of the reduced metal NPs.

The Seebeck coefficient of the micropatterns was also estimated. With double exposure at 0.030 J/cm^2^, the Seebeck coefficient of the micropatterns was 57.9 µV/K, which was smaller than that observed with single writing at 0.059 J/cm^2^. These results are listed in Table 3. This small Seebeck coefficient was induced by the generation of CuO because the temperature gradient that contributes to the thermoelectric voltage is decreased by the CuO. Therefore, the open-circuit voltage, through which we estimate the Seebeck coefficient, also decreased.

### 3.4. Test Fabrication of a Thermocouple

**Figure 7:**
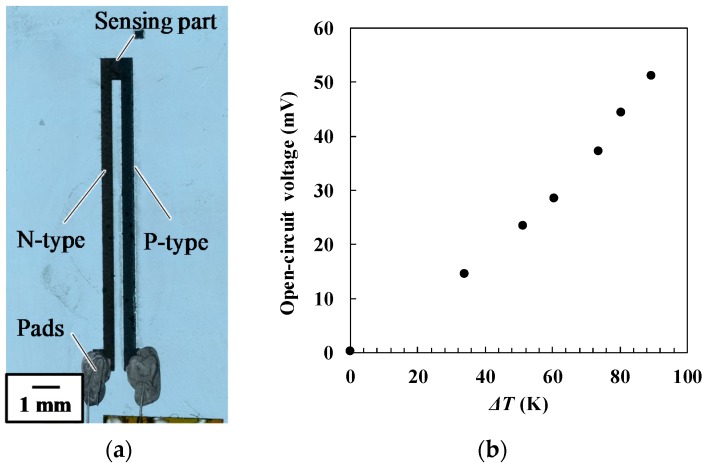
(**a**) Photograph of a sample thermocouple fabricated by selective micropatterning, (**b**) relationship between the open-circuit voltage and the temperature difference between sensing hot and cold.

We tested the above findings by fabricating a thermocouple, which comprised p-type and n-type thermoelectric elements. These elements were fabricated selectively by controlling the laser irradiation conditions. The p-type element was formed at a writing speed of 500 µm/s and a laser fluence of 0.059 J/cm^2^. The Seebeck coefficient of these p-type the micropatterns was expected to be 9.7 × 10^2^ µV/K, as shown in Table 1. The n-type element was formed at a writing speed of 4000 µm/s and the laser fluence of 0.047 J/cm^2^. The Seebeck coefficient of these n-type micropatterns was expected to be −19 µV/K, as listed in Table 3. All the elements were patterned with raster scanning of the laser focal point. The raster pitch was 10 µm, as discussed in the previous section. Figure 7a shows a photograph of the thermocouple. No damage to the micropatterns was apparent. The open-circuit voltage of the thermocouple was measured while a temperature difference between the sensing part and electrical contacts was generated. Figure 7b plots the relationship between the open-circuit voltage and the temperature difference. The voltage increased nearly linearly as the temperature difference increase. The difference from perfect linearity is possibly caused by the temperature dependence of Seebeck coefficient. A voltage of 51.2 mV was generated at the temperature difference of 89 K, which was smaller than the value of 88 mV estimated using the Seebeck coefficients of each element. The difference may be caused by the thermal history’s effect on the micropatterns while the connected region of p- and n-type thermoelectric elements in the sensing tip was being fabricated at the two different laser-writing conditions.

## 4. Discussion

We investigated effects of heat accumulation on the fabrication of micropatterns using femtosecond laser reductive sintering of the CuO/NiO NPs. Even though the total incident energy for micropatterning was constant, variations in the laser fluence and writing speed affect the content of Cu, Ni, their alloys, and oxides. In the reductive sintering of CuO/NiO NPs, first, EG is dehydrated at 433–473 K and acetaldehyde is subsequently generated [15]. Then, acetaldehyde reduces NPs mixed with CuO and NiO into Cu and Ni NPs under sufficient energy from laser irradiation [8]. After that, Cu and Ni NPs form the Cu-Ni alloy [16]. When an excess of laser energy is applied to the mixed CuO/NiO NPs, the reduced Cu and Ni become reoxidized. Temperatures above 673 K lead Cu to form CuO [8,17]. In this case, composite metal oxide micropatterns are formed. If insufficient laser energy is applied to the mixed CuO/NiO NPs, some of the original CuO and NiO material is included in the micropatterns.

Even if the incident total energy was constant, the laser fluence affected the generation of the composite materials in our tests. The maximum temperature achieved decreased with decreasing laser fluence. The heating duration also increased with slower writing speeds. As a result, the fabricated micropatterns were easily reoxidized, as shown in Figure 5.

When the laser-writing speed and the incident total energy were constant, the use of single or double exposure affected the composites in the micropatterns. The proportion of metal oxides was higher with double exposure than that with single writing as shown in Figure 6. The maximum temperature achieved with the single writing at high laser fluence was higher than that with double exposure at low laser fluence. In addition, the total laser irradiation duration with the single writing was shorter than that at with the double exposure. These factors limit the diffusion of oxygen in the air to the micropatterns with the single-writing process.

Finally, the test fabrication of a thermocouple suggests the importance of the micropattern’s thermal history. When the two micropatterns were selectively formed using two different laser irradiation conditions, each micropattern was differently thermally affected by the laser irradiation near the boundary of the two micropatterns. The composition and thermoelectric properties of the micropatterns may be controlled precisely by considering the thermal effects in the area surrounding the region of laser irradiation.

In this article, we experimentally and qualitatively investigated the patterning properties. However, the quantitative discussion is important. In the future, we will consider the phase transition using computational simulation of the temperatures.

## 5. Conclusions

We investigated the properties of micropatterns fabricated by the femtosecond laser reductive sintering of CuO/NiO mixed NPs. We found that heat accumulation during the laser irradiation process and the thermal history of the material significantly affected the composition and thermoelectric properties of the micropatterns fabricated by reductive sintering. Our tests show that selective fabrication of p-type and n-type thermoelectric micropatterns is possible with laser sintering of metal oxide NPs.

## Figures and Tables

**Table 1:** Seebeck coefficients of micropatterns printed at various writing speeds.

Writing Speed (µm/s)	Seebeck Coefficient (µV/K)	Thermoelectric Type
100	2.7 × 10^2^	p-type
500	9.7 × 10^2^	p-type
1000	0	-
3000	0	-
5000	−29	n-type

**Table 2:** Laser irradiation conditions at constant total irradiation energy.

Laser Fluence (J/cm^2^)	Writing Speed (µm/s)
0.012	1000
0.024	2000
0.035	3000
0.047	4000
0.059	5000

**Table 3:** Seebeck coefficients of the micropatterns at constant total irradiation energy and various writing speeds.

Laser Fluence (J/cm^2^)	Writing Speed (µm/s)	Seebeck Coefficient (µV/K)	Thermoelectric Type
0.012	1000	No pattern	No pattern
0.024	2000	−11	n-type
0.035	3000	−18	n-type
0.047	4000	−19	n-type
0.059	5000	−29	n-type

## References

[1] Tang Y., Loh H.T., Wong Y.S., Fuh J.Y.H., Lu L., Wang X. (2003). Direct laser sintering of a copper-based alloy for creating three-dimensional metal parts. J. Mater. Process. Technol..

[2] Zhang B., Feinech N.E., Liao H.L., Coddet C. (2013). Microstructure and magnetic properties of Fe–Ni alloy fabricated by selective laser melting Fe/Ni mixed powders. J. Mater. Sci. Technol..

[3] Sato Y., Tsukamoto M., Yamashita Y. (2015). Surface morphology of Ti-6Al-4V plate fabricated by vacuum selective laser melting. Appl. Phys. B.

[4] Dai N., Zhang L.-C., Zhang J., Zhang X., Ni Q., Chen Y., Wu M., Chao C. (2016). Distinction in corrosion resistance of selective laser melted Ti-6Al-4V alloy on different planes. Corros. Sci..

[5] Theodorakos I., Zacharatos F., Geremia R., Karnakis D., Zergioti I. (2015). Selective laser sintering of Ag nanoparticles ink for applications in flexible electronics. Appl. Surf. Sci..

[6] Watanabe A. (2013). Laser sintering of metal nanoparticle film. J. Photopolym. Sci. Technol..

[7] Kwon J., Cho H., Eom H., Lee H., Suh Y.D., Moon H., Shin J., Hong S., Ko S.H. (2016). Low-temperature oxidation-free selective laser sintering of Cu nanoparticle paste on a polymer substrate for the flexible touch panel applications. Appl. Mater. Interfaces.

[8] Kang B., Han S., Kim H.J., Ko S., Yang M. (2011). One-step fabrication of copper electrode by laser-induced direct local reduction and agglomeration of copper oxide nanoparticle. J. Phys. Chem. C.

[9] Lee H., Yang M. (2015). Effect of solvent and PVP on electrode conductivity in laser-induced reduction process. Appl. Phys. A.

[10] Lee D., Paeng D., Park H.K., Grigoropoulos C.P. (2014). Vacuum-free, maskless patterning of Ni electrodes by laser reductive sintering of NiO nanoparticle ink and its application to transparent conductors. ACS Nano.

[11] Paeng D., Lee D., Yeo J., Yoo J.H., Allen F.I., Kim I., So H., Park H.K., Minor A.M., Grigoropoulos C.P. (2015). Laser-induced reductive sintering of nickel oxide nanoparticles under ambient conditions. J. Phys. Chem. C.

[12] Mizoshiri M., Ito Y., Sakurai J., Hata S. (2016). Direct fabrication of Cu/Cu_2_O composite micro-temperature sensor using femtosecond laser reduction patterning. Jpn. J. Appl. Phys..

[13] Tamura K., Mizoshiri M., Sakurai J., Hata S. (2017). Ni-based composite microstructures fabricated by femtosecond laser reductive sintering of NiO/Cr mixed nanoparticles. Jpn. J. Appl. Phys..

[14] Mizoshiri M., Hata S. (2018). Selective fabrication of p-type and n-type thermoelectric micropatterns by the reduction of CuO/NiO mixed nanoparticles using femtosecond laser pulses. Appl. Phys. A.

[15] Fievet F., Lagier J.P., Blin B., Beaudoin B., Figlarz M. (2011). Homogeneous and heterogeneous nucleations in the polyol process for the preparation of micron and submicron size metal particles. Solid State Ion..

[16] Wada N., Kankawa Y., Kaneko Y. (1997). Comparison between Cu-Ni alloy and Cu, Ni powder mixture by metal injection molding. J. Jpn Soc. Powder Metall..

[17] Kevin M., Ong W.L., Ho G.W. (2011). Formation of hybrid structures: Copper oxide nanocrystals templated on ultralong copper nanowires for open network sensing at room temperature. Nanotechnology.

